# Everybody's Page

**Published:** 1891-08-29

**Authors:** 


### EVERYBODY'S PAGE.

An ancient and long-lost treatise on Hindoo Medicine by Banga Sen, who is quoted freely in standard works on Hindoo medicine, is reported to have been discovered recently, and to have been published.

---

It appears that the report that the camphor supplies in South Formosa are becoming exhausted is not correct. It applies only to a few purely Chinese districts, where the supply is falling off in consequence of the reckless manner in which the trees have been destroyed. Nature has kindly intervened to combat man's wastefulness by giving a practically inexhaustible supply of trees.

---

A farmer who got several innocent people into trouble has been fined for diluting his milk. This gentleman explained the presence of water in the milk in the following very disingenuous manner. His theory was that the milk was allowed to stand about in pans, and there being a lot of water about, *some of it might have got in*. This is the old story of the cat and the butter familiar to some householders, and evidently the farmer is no believer in the theory that water does not run up hill.

---

The Corporation of London are to be congratulated upon the promptitude with which they took action in remodelling and renewing the drainage system at the Freemen's Orphan School at Brixton. Thereby they probably saved the school from imminent catastrophe. There was, we fear, no novelty in the result of the inspection of the drainage. Of how many houses in our large towns could it be said with strict accuracy and truth that the system of drainage is not inefficient, and in many cases not antiquated, or a source of serious danger to the inhabitants.

---

Vinegar is regarded by an American physician, Dr. S. J. Bumstead, as a valuable therapeutic agent in catarrhal and membranous croup. Employed in the form of inhalation, it is, he considers, of first importance in the management of the disease, though he also employs internal medication. His method of procedure in cases of inhalation is to pour the vinegar into a pan, and then put in the pan bricks or flat irons heated in the stove. The room thus soon becomes filled with a cloud of acetic vapour. A German doctor reports the use of etherisation with good results in the case of a child aged thirteen months who was apparently [illegible] when he was called in.

